# Short-Wave Infrared Optoelectronics with Colloidal CdHgSe/ZnCdS Core/Shell Nanoplatelets

**DOI:** 10.1021/acsphotonics.4c01944

**Published:** 2024-12-19

**Authors:** Hossein Roshan, Anatol Prudnikau, Jinfei Dai, Matilde Cirignano, Francesco De Boni, Mirko Prato, Fabian Paulus, Vladimir Lesnyak, Francesco Di Stasio

**Affiliations:** † Photonic Nanomaterials, Istituto Italiano di Tecnologia, 16163 Genova, Italy; ‡ Leibniz-Institute for Solid State and Materials Research (IFW) Dresden, Helmholtzstrasse 20, 01069 Dresden, Germany; § Key Laboratory for Physical Electronics and Devices of the Ministry of Education & Shaanxi Key Lab of Information Photonic Technique, School of Electronic Science and Engineering, Xi’an Jiaotong University, Xi’an 710049, China; ∥ Materials Characterization Facility, Istituto Italiano di Tecnologia, Via Morego 30, 16163 Genova, Italy; ⊥ Center for Advancing Electronics Dresden (cfaed), TU Dresden, Helmholtzstrasse 18, 01069 Dresden, Germany; ○ Physical Chemistry, TU Dresden, Zellescher Weg 19, 01069 Dresden, Germany

**Keywords:** colloidal semiconductor, nanocrystals, nanoplatelets, short-wave infrared, near-infrared, light-emitting diode, photodiode

## Abstract

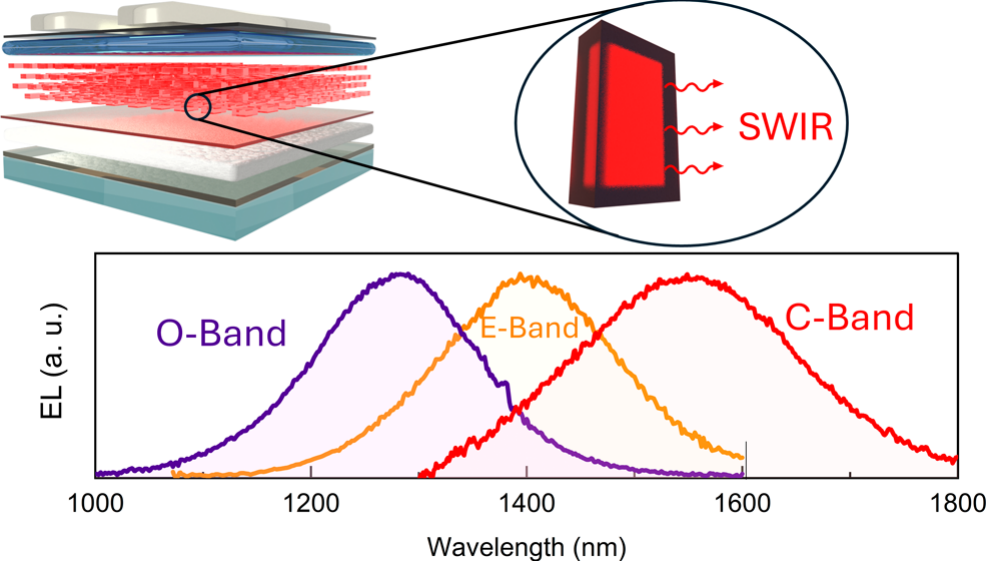

Colloidal semiconductor nanocrystals (NCs) are an efficient and cost-effective class of nanomaterials for optoelectronic applications. Advancements in NC-based optoelectronic devices have resulted from progress in synthetic chemistry, adjustable surface properties, and optimized device architectures. Semiconductor nanoplatelets (NPLs) stand out among other NCs due to their precise growth control, yielding uniform thickness with submonolayer roughness. In this study, we demonstrate the versatility of core/shell Cd_*x*_Hg_1–*x*_Se/Zn_*y*_Cd_1–*y*_S NPLs for optoelectronic applications in the short-wave infrared (SWIR) spectral range. We employed the very same core/shell NPLs for the fabrication of light-emitting diodes and photodetectors alike, achieving significant performance in both electroluminescence (external quantum efficiency ranging from 7.5% at 1280 nm to 3.8% at 1550 nm) and detection (responsivity of 0.24 A W^–1^ at 1200 nm).

## Introduction

In the field of optoelectronic systems, short-wave infrared (SWIR, 800–1700 nm) light presents an opportunity to acquire information and transmit it in an invisible spectral range for the human eye, which is sensitive from ≈ 350 to ≈ 700 nm.^1^ SWIR optoelectronics plays a pivotal role in active imaging of living tissues, owing to distinctive attributes like diminished scattering and absorption, substantial penetration depth in tissues,^2^ and minimized phototoxicity.^1,3^ SWIR active imaging is also applicable in industrial machine vision, moisture detection, and materials sorting.^4^ Fiber-optic and free space optical communication (FOC and FSO, respectively) systems are other important applications of SWIR technology. In fact, both FOC and FSO take advantage of the SWIR spectral range, where both optical fibers and atmosphere show low attenuation and scattering, thus, extending the transmission path distance with minimal risk for the human eye.^5,6^

All these applications require efficient light sources and detectors, which are traditionally provided by epitaxial technology, predominantly relying on indium arsenide (InAs) or indium gallium arsenide (InGaAs).^7^ Yet, epitaxial semiconductor technology caters mainly to FOC networks and only a few other applications due to constraints, such as difficult scalability, high production costs, and incompatibility with complementary metal-oxide semiconductor (CMOS) technology.^8^ Conversely, colloidal semiconductor nanocrystals (NCs) present a more promising avenue for SWIR optoelectronics, offering advantages, such as cost-effectiveness, scalability, and CMOS compatibility.^9^ Recent strides in narrow bandgap semiconductor compositions and solution-processing techniques offer a promising trajectory toward the realization of high-performance SWIR optoelectronic systems based on NCs, also owing to the considerable versatility of their optical properties and desirable characteristics, among which are high absorption coefficients and photoluminescence quantum yields (PLQYs). The efficacy of such technology hinges on the performance of light sources and photodetectors in this spectral range. Nonetheless, despite the commendable results of NC-based LEDs in the visible spectrum, with external quantum efficiencies (EQEs) exceeding 20%,^10^ their SWIR counterparts have shown much diminished performance.^11^

Among SWIR NCs, PbS NCs are the most developed in terms of synthesis protocols,^12^ optical tunability,^13^ electrical transport,^14^ and device applications.^15−17^ While the concept of optical communication links based on colloidal optoelectronic devices had been previously investigated in the visible spectral range,^18^ optical communication in the SWIR range remained unexplored until 2022, when Pradhan et al. exploited PbS NCs for implementing such applications. The authors fabricated an LED with stacked layers of ITO/n-ZnO/PbS NCs blended with ZnO/p-PbS NCs/Au exhibiting an EQE of 11.8% at 1550 nm^19^ and 7.9% at 1400 nm.^20^ Importantly, they used the same structure as a photodiode with a responsivity peak of 0.21 A W^–1^ at 1560 nm.^19^ In a similar work, Qu et al. used HgTe NC-LEDs coupled with an infrared camera, demonstrating an active imaging setup. Thanks to the size tunability of HgTe NCs, the fabricated LEDs showed electroluminescence (EL) starting from 1100 to >1700 nm, covering the SWIR spectral range with a peak EQE of 0.67%,^4^ which later was optimized to 2.2%^21^. Among all various compositions, Hg/Cd chalcogenide alloys offer the advantage of bandgap tunability by adjusting the Hg to Cd ratio.^22^ Alloying enables the detection of wavelengths spanning from 0.8 to 15 μm (by tuning the Hg:Cd ratio from 0:100 to 95:5). Building upon this foundation, it became feasible to extend the emission of CdSe nanoplatelets (NPLs) into the SWIR by substituting Cd cations with Hg, yielding Cd_*x*_Hg_1–*x*_Se.^23^ In our previous work,^24^ we have demonstrated the synthesis of NIR-emitting Cd_*x*_Hg_1–*x*_Se/Zn_*y*_Cd_1–*y*_S core/shell NPLs via a cation-exchange method, starting from four monolayer (ML) thick CdSe NPLs, followed by the shell growth. The obtained core/shell NPLs were employed in LEDs, achieving an EL at 1300 nm with a peak EQE of 7.5%. We have also shown that the lateral size of these NIR-emitting NPLs affects their PL spectral position, probably due to the presence of lateral exciton confinement, in addition to the strong confinement in the thickness direction.

In this work, we prepared colloidal Cd_*x*_Hg_1–*x*_Se/Zn_*y*_Cd_1–*y*_S core/shell NPLs with an emission peak between 1297 and 1566 nm using identical protocols for cation exchange and shell growth, varying only the lateral size of the starting 4 ML thick CdSe NPLs. The NPLs were used as active layers in LEDs, leading to EL in the SWIR region with an EQE up to 7.5%. Moreover, we demonstrate that the same core/shell NPLs can be used as the absorbing layer of a photodiode with a peak EQE of 25% at 1200 nm.

## Results and Discussion

**Figure 1:**
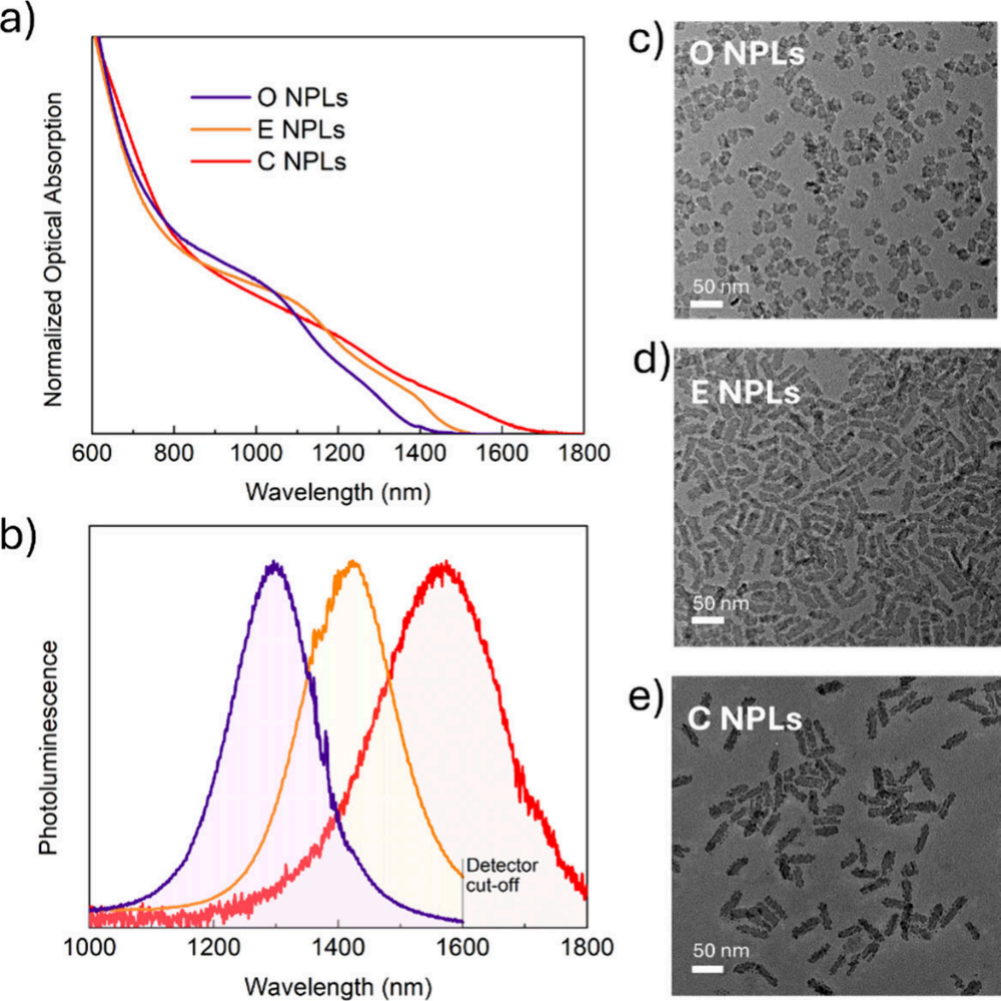
(a) Normalized optical absorption and (b) PL spectra of the three Cd_*x*_Hg_1–*x*_Se/Zn_*y*_Cd_1–*y*_S core/shell NPL samples (O, E, and C) in TCE dispersions. TEM images of (c) O, (d) E, and (e) C NPLs.

Three distinct Cd_*x*_Hg_1–*x*_Se/Zn_*y*_Cd_1–*y*_S core/shell NPL samples were synthesized employing the methodology developed in our previous study.^24^ The synthetic procedure includes partial exchange of Cd^2+^ to Hg^2+^ cations in 4 ML thick CdSe NPLs and coating of the obtained Cd_*x*_Hg_1–*x*_Se NPLs with a thick Zn_*y*_Cd_1–*y*_S shell. For cation exchange, we used CdSe NPLs with different initial lateral sizes (Figure S1 in Supporting Information), while all other parameters, including the ratio between CdSe and the amount of the added mercury precursor, remained the same for all samples. The optical absorption and PL spectra,and the TEM images of the three samples are presented in Figure 1. NPLs exhibit tunability of their bandgaps across the SWIR spectrum, as evidenced by the optical absorption edge ranging from ≈1400 to 1700 nm (Figure 1a), and PL between 1100 and 1800 nm (Figure 1b, the PL from compact films made of the same NPLs is shown in Figure S2). The three samples used in this study, with EL peaks at 1280, 1400, and 1550 nm, are referred to as O, E, and C NPLs, corresponding to the standard O-band (original band: 1260–1360 nm), E-band (extended-wavelength band: 1360–1460 nm), and C-band (conventional band 1530–1565 nm) in FOC networks. All three samples exhibit a PLQY ranging from 50 ± 5 to 58 ± 6% in tetrachloroethylene (TCE) dispersions and from 30 ± 3 to 39 ± 4% in solid films on glass, in line with our previous report.^24^ Samples O, E, and C show PL peaks at 1297 (0.956 eV), 1416 (0.876 eV), and 1566 nm (0.792 eV) with a full width at half-maximum (fwhm) of 159 (0.118 eV), 180 (0.112 eV), and 241 nm (0.123 eV), respectively, in TCE dispersion; and at 1335 (0.929 eV), 1444 (0.859 eV), and 1608 nm (0.771 eV) with a fwhm of 160 (0.112 eV), 180 (0.107 eV), and 262 nm (0.126 eV), respectively, in solid films on glass. The PL in films is red-shifted due to the close packing of NPLs, which enables energy transfer;^25^ however, the dispersion and solid film of each sample show similar fwhm values. All the emission properties of the O, E, and C NPLs are summarized in Table S1 of the Supporting Information. Figure 1c–e shows TEM images of the 3 different core/shell NPL samples. The average sizes of the O, E, and C NPLs are 21.8 × 16.4, 39.0 × 14.9, and 45.0 × 15.4 nm^2^, respectively. We also performed X-ray photoelectron spectroscopy (XPS) measurements on each sample (Figures S3–S5). XPS spectra confirm the presence of all the expected elements. Moreover, the binding energy of Zn 2p, Cd 3d, S 2p, Hg 4f, and Se 3d peaks agrees with the expected chemical state for each element in the NPLs (i.e., Zn^2+^, Cd^2+^, Hg^2+^, S^2–^, Se^2–^).

**Figure 2:**
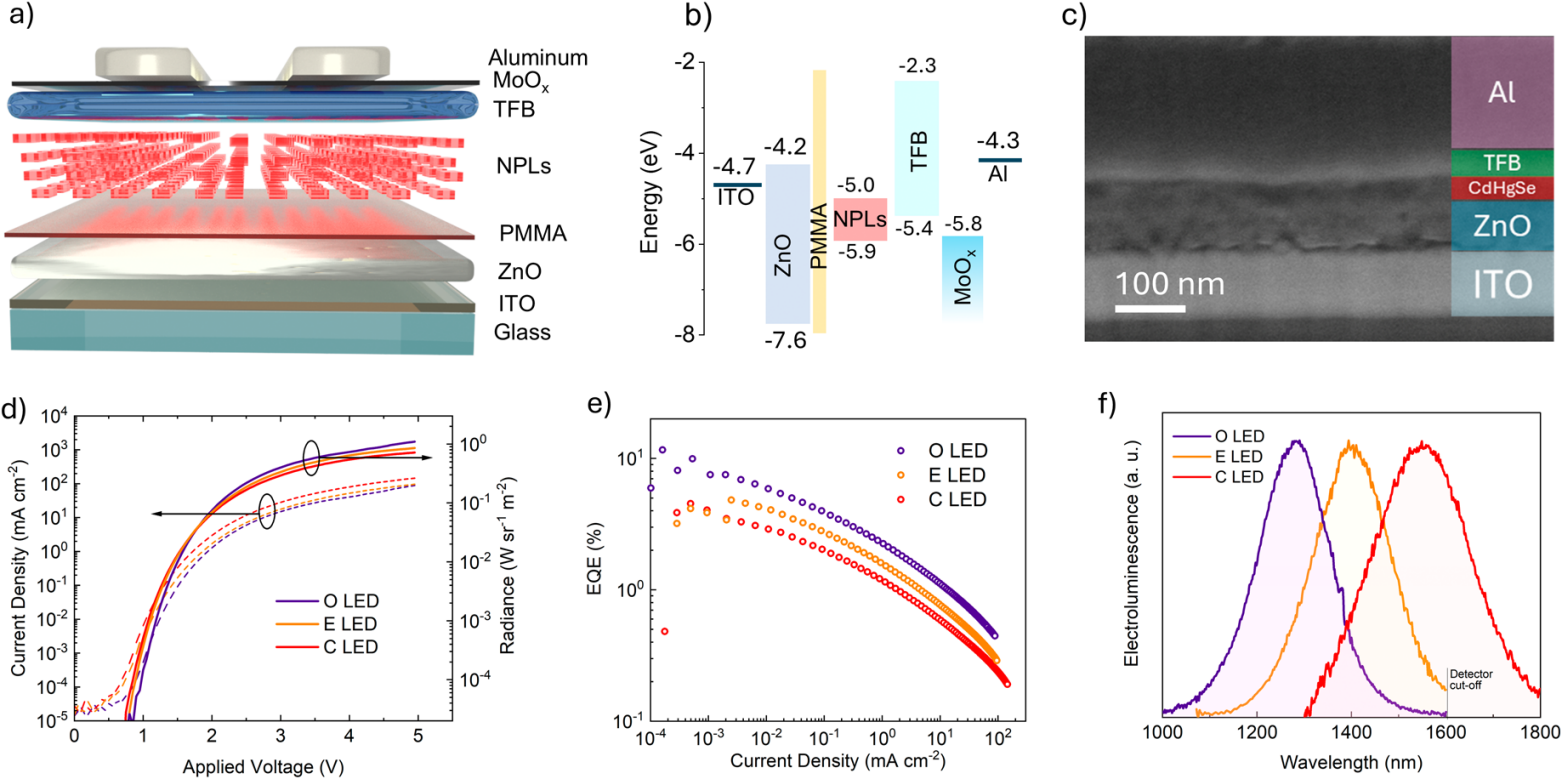
(a) Schematic of the LED architecture. (b) Flat energy band diagram of the LED. (c) SEM cross-sectional image of a typical LED. (d) J-V-R curves of the three types of LEDs. (e) EQE versus current density curves. (f) EL spectra of LEDs with different active layers.

We fabricated LEDs incorporating the three different NPL samples employing the same device structure we recently published for Cd_*x*_Hg_1–*x*_Se/Zn_*y*_Cd_1–*y*_S core/shell NPLs with an emission at 1300 nm.^24^ We developed such architecture by testing a variety of commercially available transport layers and electrodes with different work functions. We successfully employed such hybrid organic/inorganic device structures also for InAs/ZnSe NC-LEDs.^26^ The LEDs were produced by initially establishing an electron transport layer (ETL) using sol–gel ZnO, accompanied by a thin film of poly(methyl methacrylate) (PMMA), which functions as a modifier to enable precise control over electron injection. This film serves as an insulating layer that optimizes the charge balance by regulating electron injection. Also, by reducing excess electron currents, the layer preserves the luminescent properties of NCs, thus leading to improved stability in addition to enhanced efficiency.^27^ The hole transport layer (HTL) relies on poly(9,9-dioctylfluorene-*alt*-*N*-(4-s-butylphenyl)-diphenylamine) (TFB) due to its superior band alignment, resulting in a decreased turn-on voltage and optimal charge balance within the active layer, thereby enhancing the EQE. Figure 2a shows a schematic of the LED, presenting all layers, while Figure 2b illustrates the flat energy band diagram of the structure embedding C NPLs. Energy levels of the various layers and electrodes were obtained from literature,^28−31^ while ultraviolet photoelectron spectroscopy (UPS) spectra of the three NPL samples are provided in the Supporting Information (see Figures S3–S5). The UPS spectra do not show a distinct difference between NPLs in terms of valence band maximum (VBM), which is at −5.9 ± 0.2 eV for all three samples. It is important to consider that the energy variations across the three NPL samples are small, e.g., the energy bandgap changes from ≈0.9 eV (O NPL sample) to ≈0.8 eV (C NPL sample, all values estimated from the Tauc plots in Figure S6). If we consider that the error on the energy values from UPS analysis is 0.1–0.2 eV, then it is evident that VBM or conduction band minimum (CBM) variations across the samples cannot be clearly resolved. Therefore, in the flat energy band diagram, we report the CBM and VBM for the O NPL sample, for the sake of clarity.

The scanning electron microscopy (SEM) cross-sectional image displays the prepared stack layers of a typical LED (Figure 2c), where ITO (100 nm), ZnO (70 nm), Cd_*x*_Hg_1–*x*_Se/Zn_*y*_Cd_1–*y*_S NPLs (25 nm), TFB (30 nm), MoO_*x*_ (10 nm), and an aluminum contact (approximately 150 nm) are distinctly visible. Figure 2d illustrates the current density and radiance of LEDs versus the applied voltage (J-V-R). These curves display a low turn-on voltage ranging from 0.8 to 0.9 V, closely aligning with the bandgap energy of the respective emissive layer. Maximum radiance was observed ranging from 0.8 to 1.5 W sr^–1^ m^–2^. Although the maximum radiance obtained is relatively low, the current density is also considerably small, thus it results in a relatively high EQE.^4,18^ EQE as a function of current density for various wavelengths is shown in Figure 2e. A maximum EQE of around 7.5% is observed for the O LED (fabricated with O NPLs) at a current density of approximately 1 mA cm^–2^ (EL peak = 1280 nm). The maximum EQE for the E and C LEDs is 4.8% and 3.8%, with EL peaks at 1400 and 1550 nm, respectively. The roll-off in EQE with increasing current density is approximately one order of magnitude and is similar for all devices investigated. Such a drop in EQE can be tentatively attributed to increased charge imbalances in the active layer at a high current density. In Figure S7, the power conversion efficiency (PCE) of the LEDs is presented versus the current density. At the maximum applied current, achieved with a forward bias of 5 V and a current density of 0.1 A cm^–2^, the devices demonstrate EQEs ranging from 0.4% to 0.2% in Figure 2e. Correspondingly, at this point, the PCE varies from 0.08% to 0.03% across the various devices. While the EQE experiences a noticeable decrease as the current increases, the observed decline in PCE is even more pronounced. This significant drop in PCE in comparison with EQE at higher current levels is primarily attributed to ohmic losses within the ITO electrode. These ohmic losses, which increase with current density, exacerbate the efficiency drop by power conversion to heat (power loss), which would otherwise contribute to the optical output.^32^ The EL spectra of all LEDs are shown in Figure 2f. A slight blue-shift with respect to the PL from solid films (Figure S2) is observed for all samples. An interesting aspect of these LEDs (O, E, and C) is the observable trend in the EQE; specifically, we found that the maximum EQE decreases from 7.5% at 1280 nm to 3.8% at 1550 nm. Such a drop in EQE is surprising, given that all samples show PLQY in film on glass ranging from 39% to 30% (Table S1). However, we can tentatively assign this reduction in EQE to a combination of different effects, such as increased optical absorption by the ITO,^33,34^ variations in charge balance in the active layer and different optical outcoupling efficiencies for the emitted light.

To further highlight the versatility of Cd_*x*_Hg_1–*x*_Se/Zn_*y*_Cd_1–*y*_S NPLs, we tested their properties in a photodiode structure. In fact, using the same active nanomaterial for different types of optoelectronic devices can be highly cost-effective, as it streamlines fabrication by focusing on a single synthesis process for both applications. It should be noted that achieving a high PLQY, which is essential for efficient LEDs, requires shell growth.^35^ While shell growth is beneficial for PLQY and LED performance, core/shell architectures can be detrimental to photodetectors and solar cells, as the shell may impede charge extraction from the core.^9,36−38^

**Figure 3:**
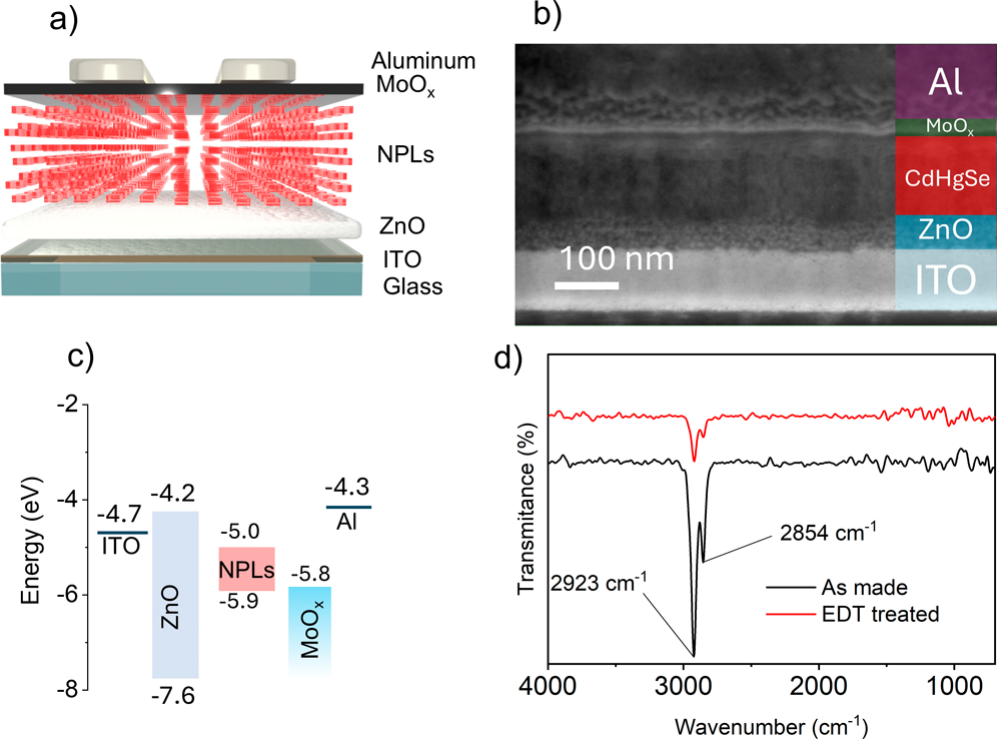
(a) Schematic, (b) cross-section SEM image, and (c) flat band diagram of a Cd_*x*_Hg_1–*x*_Se/Zn_*y*_Cd_1–*y*_S NPL photodiode. (d) FTIR spectra of a Cd_*x*_Hg_1–*x*_Se/Zn_*y*_Cd_1–*y*_S NPL film before and after ligand exchange with EDT.

The photodiode structure was inspired by a related study^39^ and is shown in Figure 3a, with an SEM cross-section image in Figure 3b and the flat band energy diagram in Figure 3c. For the fabrication of a photodiode, we used the C NPL sample, thus enabling optical absorption over a large portion of the SWIR spectral range. A ZnO layer was used as the ETL and MoO_*x*_ as the HTL, the former facilitates the electron extraction from the NPLs, while the latter enables hole extraction, thus leading to photocurrent generation even with zero bias voltage. To increase the optical absorption of the active layer, a thick film of C NPLs was deposited using layer-by-layer spin-coating^40^ and ligand exchange with 1,2-ethanedithiol (EDT). Typically, long-chain fatty acids or amines, such as oleic acid (OlAc) or oleylamine, are utilized in the NC’s synthesis process as ligands, controlling the growth and forming a compact organic shell attached to the surface, ensuring colloidal stability. However, these long-chain ligands hinder the carrier transport between NCs. As a result, they are often substituted with short-chain ligands to reduce the interparticle distance. Short-chain thiol molecules are commonly used as capping ligands given that their –SH headgroup strongly binds to surface cations, displacing the carboxyl headgroup of the fatty acid (details of the fabrication and ligand exchange are available in the experimental section).^41^ In our case, we performed a solid-state ligand exchange with EDT which we verified via Fourier transform infrared (FTIR) spectroscopy. Figure 3d displays two FTIR spectra of the same NPL film before and after treatment with EDT ligands (originally capped with OlAc). The presence of OlAc in the pristine film is confirmed by the two characteristic peaks at 2923 cm^–1^ (asymmetric C–H) and 2854 cm^–1^ (symmetric C–H). In the treated film, the reduction in amplitude of these two peaks confirms the successful ligand exchange and displacement of OlAc (due to the stronger thiol–metal bonds formed during the exchange, which surpass the strength of carboxyl–metal bonds).^42^

**Figure 4:**
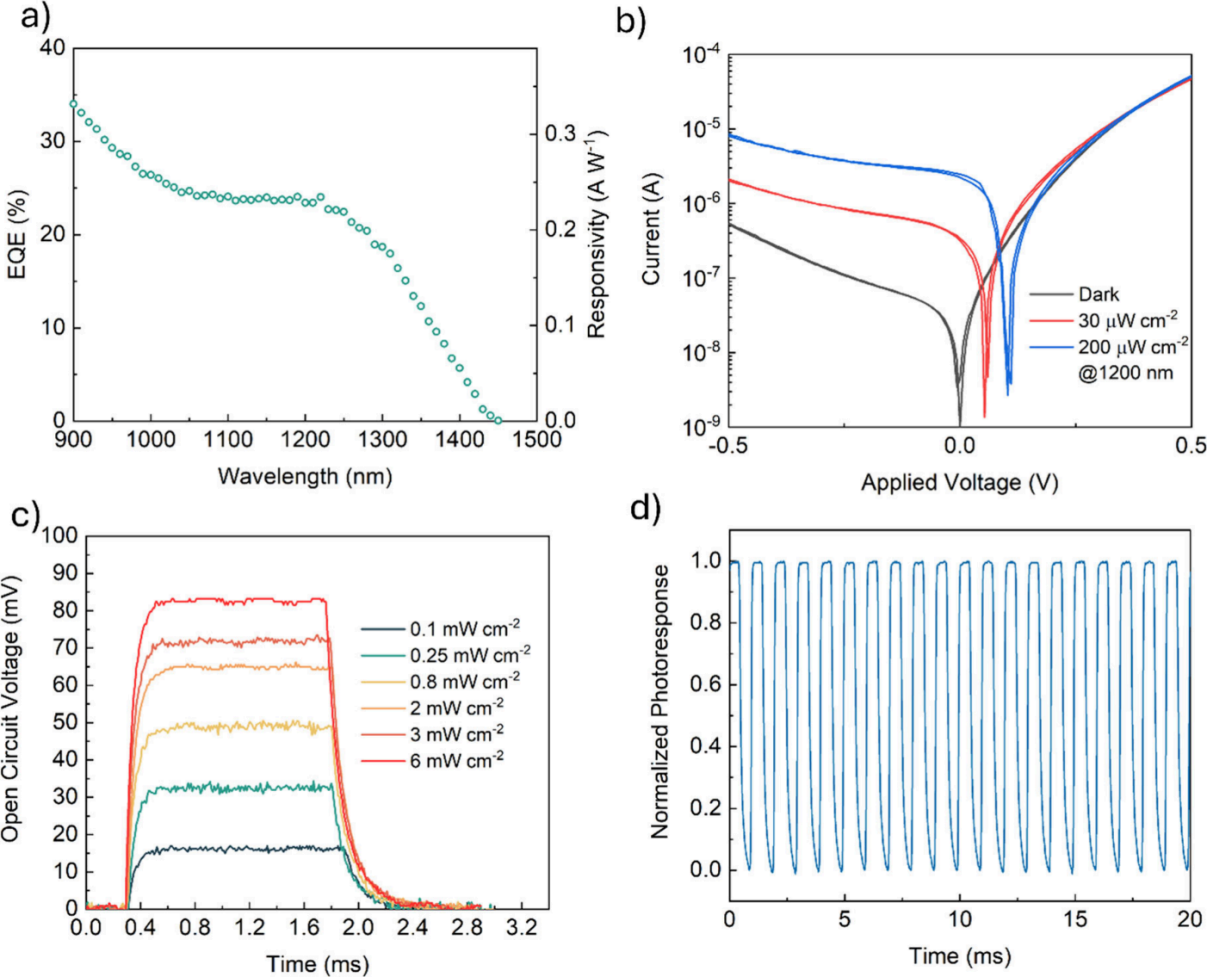
(a) Response spectrum, (b) *J*–*V* curves, (c) open circuit voltage response to 1200 nm light, and (d) reproducibility of the response to 1200 nm light pulses of the fabricated photodiode.

Figure 4a (known as spectral response) illustrates the EQE and responsivity of the photodiode across the SWIR spectrum by utilizing the C NPLs. A distinct shoulder at approximately 1200 nm indicates the first excitonic transition with an EQE of 25% and a responsivity of 0.24 A W^–1^. These values are compared with those of state-of-the-art photodiodes working in the 900–1400 nm range in Table S2. The obtained photodiode demonstrates superior performance when compared to recent ones compliant with the “Restriction of Hazardous Substances in Electrical and Electronic Equipment” directive (RoHS).^9,43^ However, it exhibits diminished performance with respect to state-of-the-art PbS NC photodiodes,^44^ and it might be caused by the thick shell of NPLs, which hinders the efficient charge extraction from the Hg-containing core. The current–voltage (*I*–*V*) characteristics (Figure 4b) show that the photodiode provides a photoresponse to incident light intensity (λ = 1200 nm) as low as 30 μW cm^–2^, affirming the efficacy in detecting weak optical signals. Additionally, it displays diode behavior in dark conditions, as the current density increases exponentially in forward bias, and a saturation current is observed in reverse bias. The I–V curves also exhibit a lack of hysteresis. The transient response is shown in Figure 4c, and it was investigated by illuminating the photodiode with pulsed 1200 nm light with different optical power density. For this purpose, the open circuit voltage (with a large impedance, 10^6^ ohms) of the photodiode was measured, and it exhibits a prolonged fall time attributable to the high resistance–capacitance (*R*·*C*) time constant of the device and measurement system. Figure S8 shows the open circuit voltage of the photodiode in response to 1200 nm light, while Figure 4d elucidates the reproducibility of the response to a constant pulsed light with the transmitter-receiver distance set at 5 cm.

## Conclusions

In summary, we have designed SWIR-emitting LEDs with peak emissions in O, E, and C optical telecom bands utilizing tunable core/shell Cd_*x*_Hg_1–*x*_Se/Zn_*y*_Cd_1–*y*_S NPLs. These NPLs can be employed also for the fabrication of photodiodes, despite the presence of a thick shell. The obtained LEDs and photodiodes can potentially be used in applications, such as active imaging and telecommunication systems, which currently depend on epitaxial technology. The LEDs demonstrated a peak EQE of 7.5%, while the photodiodes achieved an EQE of 25% at a wavelength of 1200 nm.

## Experimental Methods

### Materials

Zinc acetate dihydrate (Zn(OAc)_2_·2H_2_O, 99%), 2-methoxyethanol, ethanolamine, poly(methyl methacrylate) (PMMA), chlorobenzene (99.8%), ethanol (99.8%), acetone (99.8%), and acetonitrile (99.9%) were purchased from Sigma-Aldrich. Molybdenum oxide (MoO_3_) was acquired from Alpha Chemicals; poly(9,9-dioctylfluorene-*alt*-*N*-(4-*s*-butylphenyl)-diphenylamine) (TFB) was purchased from Ossila.

### Synthesis of Cd_*x*_Hg_1–*x*_Se/Cd_*y*_Zn_1–*y*_S Core/Shell NPLs

Cd_*x*_Hg_1–*x*_Se/Cd_*y*_Zn_1–*y*_S NPLs were synthesized by the procedure we reported previously in ref (24). Details of the synthesis are outlined in the Supporting Information.

### Characterization

Optical absorption was measured by using a UV-vis-NIR spectrophotometer (Cary 5000, Varian). PLQY measurements were performed with an Edinburgh Instruments FLS900 spectrofluorometer equipped with integrating spheres and excited at 1000 nm using a Xe lamp. TEM imaging was conducted on a Jeol JEM F200 instrument at 200 kV.

### X-ray Photoelectron Spectroscopy (XPS) and Ultraviolet Photoelectron Spectroscopy (UPS)

XPS measurements were carried out through a Kratos Axis Ultra^DLD^ spectrometer (Kratos Analytical Ltd.) with a monochromated Al Kα X-ray source (*h*ν = 1486.6 eV) operating at 20 mA and 15 kV. Specimens were prepared by spin-coating a concentrated NPL solution onto a silicon substrate.

The wide scans were collected over an analysis area of 300 × 700 μm^2^ at a photoelectron pass energy of 160 eV and an energy step of 1 eV, while high-resolution spectra were collected at a photoelectron pass energy of 20 eV and an energy step of 0.1 eV. A takeoff angle of 0° with respect to sample normal direction was used for all analyses. The slight differential electrical charging effects (less than 0.5 eV) observed on all samples were not neutralized. The spectra have been referenced to the adventitious carbon 1s peak at 284.8 eV. The spectra were analyzed with the CasaXPS software (Casa Software Ltd., version 2.3.24),^45^ and the residual background was eliminated by the Shirley method across the binding energy range of the peaks of interest.

The UPS measurements were performed employing a He I (21.22 eV) discharge lamp, fitted in the same chamber used for XPS analyses, on an area of 55 μm in diameter, at a pass energy of 10 eV and with a dwell time of 100 ms. A −9.0 V bias was applied to the sample to precisely determine the low-kinetic-energy cutoff.

The energy value of the VBM versus the vacuum level, i.e., the ionization energy, *E*_ion_, was determined from the width of the entire UPS spectrum, according to the following equation: *E*_ion_ = *h*ν – (*E*_0_ – *E*_1_) = *h*ν – *E*_0_ + *E*_1_, where *h*ν is the source energy (21.22 eV for He I photons), *E*_0_ is the secondary electron cut off, and *E*_1_ is the low binding energy onset.^46^ The *E*_0_ and *E*_1_ values were determined from the UPS spectrum through the background functions “Edge Up” and “Edge Down”, respectively, in CasaXPS software. The error bar associated with this procedure was estimated to be in the 0.1–0.2 eV range.

### LED Fabrication

LEDs were fabricated using prepatterned ITO glasses and a sol–gel method for the ZnO layer. A sol–gel solution was prepared by dissolving 1.6 g of Zn(OAc)_2_·2H_2_O and 100 μL of ethanolamine in 5 mL of 2-methoxyethanol, which was stirred overnight. 30 μL of the solution was spin-coated at 4000 rpm for 50 s on the ITO glass, followed by annealing at 200 °C for 20 min. The substrates were transferred to a nitrogen-filled glovebox, where a thin PMMA layer was deposited by spin-coating a 1.5 mg mL^–1^ acetone solution of PMMA at 2000 rpm and annealed at 90 °C for 10 min. The active layer dispersion in chloroform (10 mg mL^–1^) was deposited at 1500 rpm and annealed at 80 °C. A hole-transporting layer (∼20 nm) was formed using a 14 mg mL^–1^ TFB solution in chlorobenzene, spin-coated at 2000 rpm, and annealed at 80 °C. The top electrode was deposited by using thermal evaporation of 10 nm MoO_3_ and 150 nm aluminum at 2 × 10^–6^ mbar. The active area of each device was 4.5 mm^2^.

### LEDs Characterization

The present investigation involved the characterization of devices having an area of 4.5 mm^2^. This characterization includes analysis of their current density and radiance concerning the applied bias. The measurements were carried out in an ambient air environment with a relative humidity of approximately 50%. To apply the bias and measure the current passing through the device, we utilized a Keithley 2636 source meter, which was connected to the LED. The emitted light was quantified by using a calibrated Gentec germanium-based photodetector (model PH20-Ge-D0). Radiance (*R*) was determined using the formula  (expressed in units of W sr^–1^ m^–2^), where *S*_2_ represents the emissive area, and *L*_0_ signifies the radiant intensity, denoting the power of light emitted from a source within a solid angle unit. Given that the source followed a Lambertian profile, we employed the following equation:  (in units of W sr^–1^). In this equation, *P* represents the emitted power in the forward direction perpendicular to the LED surface, and  is the solid angle between the emitting area and the photodetector (where *l*_2_ indicates the distance between them, and *S*_1_ refers to the photodetector’s area).

The EQE was determined as the ratio of the number of emitted photons (*N*_p_) in the forward direction in free space to the number of injected charge carriers (*N*_e_): EQE = *N*_p_/*N*_e_. To calculate *N*_p_, we used the formula , where λ represents the emission wavelength, *hc* is the product of the Planck constant (*h* = 6.626 × 10^–34^ J s) and the speed of light in vacuum (*c* = 3 × 10^8^ m s^–1^). The injected charge carrier *N*_e_ was obtained as , where *I* represents the current flowing into the device, and *q* is the elementary charge constant (1.6 × 10^–19^ C).

### Photodiode Fabrication

Photodiode was fabricated using prepatterned ITO glasses and a sol–gel method for the ZnO layer deposition. A sol–gel solution was prepared by dissolving 1.6 g of Zn(OAc)_2_·2H_2_O and 100 μL of ethanolamine in 5 mL of 2-methoxyethanol followed by stirring overnight. 30 μL of the solution was spin-coated at 4000 rpm for 50 s on the ITO glass, followed by annealing at 200 °C for 20 min. The substrates were transferred to a nitrogen-filled glovebox. NPL ink was prepared by dispersing 10 mg of NPLs in 1 mL of chloroform. The NPLs were spin-coated onto the ZnO/ITO substrate at 2000 rpm. This process was repeated seven times to obtain a desirable thickness, and after each NPL layer was deposited, substrates were dipped into 2% EDT solution in acetonitrile for 3 min and cleaned with pure acetonitrile. Finally, a 20 nm layer of MoO_*x*_ was deposited as the HTL from a MoO_3_ source, and a 120 nm layer of Al was thermally evaporated as the back electrode and reflector. The effective area of the photodiode was 4.5 mm^2^.

### Photodiode Characterization

The current–voltage (*I*–*V*) characteristics were recorded using a Keithley 2400 source meter under dark conditions with voltages from −0.5 to +0.5 V. To obtain EQE spectra, the devices were exposed to monochromatic light produced by a 400 W xenon lamp through a monochromator with long-pass filter with 800 nm cutoff wavelength to filter the second-order diffraction in the visible spectrum. This setup was synchronized with the Keithley device to measure the short-circuit current simultaneously. The output power was calibrated using a Newport 838-IR photodetector. Responsivity was defined as  and EQE was defined as , in which *I*_λ_ is the photocurrent (the current generated by the device) as a function of wavelength λ, and *P*_λ_ is the optical power incident on the device in wavelength of λ. *q* is the elementary charge, approximately 1.6 × 10^–19^C. *h* is Planck’s constant, approximately 6.626 × 10^–34^ J s, *c* is the speed of light in a vacuum, approximately 3 × 10^8^ m s^–1^. λ is the wavelength of the incident light in nanometers.

## Supporting Information Available

Additional morphological, optical, and electrical data; XPS and UPS data and analysis; Table reporting the performance of various photodiodes for comparison (PDF)
