# Supplementary material for: Short-Wave Infrared Optoelectronics with Colloidal CdHgSe/ZnCdS Core/Shell Nanoplatelets
Source: ACS Photonics. 2024 Dec 19;12(1):40–7. doi: 10.1021/acsphotonics.4c01944 (PMC11741132; doi:10.1021/acsphotonics.4c01944)
Supplement: Supplementary file 1 — ph4c01944_si_001.pdf [file ph4c01944_si_001.pdf]

Supporting Information

Short-wave infrared optoelectronics with colloidal CdHgSe/ZnCdS core/shell nanoplatelets

Hossein Roshan,[1,†] Anatol Prudnikau,[2,†] Jinfei Dai,[3] Matilde Cirignano,[1] Francesco De Boni,[4] Mirko Prato,[4] Fabian Paulus,[2,5] Vladimir Lesnyak,[6,*] Francesco Di Stasio[1,*]

[1] *Photonic Nanomaterials, Istituto Italiano di Tecnologia, 16163, Genova, Italy*

[2] *Leibniz-Institute for Solid State and Materials Research (IFW) Dresden, Helmholtzstrasse 20, 01069 Dresden, Germany*

[3] *Key Laboratory for Physical Electronics and Devices of the Ministry of Education & Shaanxi Key Lab of Information Photonic Technique, School of Electronic Science and Engineering, Xi'an Jiaotong University, Xi'an, 710049, China*

[4] *Materials Characterization Facility, Istituto Italiano di Tecnologia, Via Morego 30, 16163 Genova, Italy*

[5] *Center for Advancing Electronics Dresden (cfaed), TU Dresden, Helmholtzstrasse 18, 01069 Dresden, Germany*

[6] *Physical Chemistry, TU Dresden, Zellescher Weg 19, 01069 Dresden, Germany*

*Corresponding Authors: francesco.distasio@iit.it, vladimir.lesnyak@tu-dresden.de

†These authors contributed equally

NANOPLATELETS PREPARATION

Materials

Cadmium acetate ($Cd(OAc)_2$, 99.995%), mercury (II) acetate ($Hg(OAc)_2$, 98.0%), zinc acetate dihydrate ($Zn(OAc)_2 \cdot 2H_2O$, 99%), 1-octadecene (ODE, 90%), myristic acid (98.5%), oleic acid (OlAc, 90%), oleylamine (OlAm, 98%), ethanol (99.8%), and zinc diethyldithiocarbamate ($Zn(DDTC)_2$, 97%) were purchased from Sigma–Aldrich. Hexane (95%), chloroform (98.8%), acetonitrile (99.9%), methanol (99.8%), and isopropanol (IPA, 99.8%) were purchased from Fisher Chemical. Selenium powder (Se,

mesh 160, 99.99%) and cadmium acetate dihydrate ($Cd(OAc)_2 \cdot 2H_2O$, 99.99%) were purchased from Chempur. To purify $Zn(DDTC)_2$, it was dissolved in chloroform at room temperature (20 g in 200 mL) and filtered to remove all insoluble substances followed by solvent evaporation. The resulting powder was dried under vacuum overnight. All other reagents were used as received without further purification.

Synthesis of 4 ML CdSe NPLs[1,2]

A mixture of 640 mg (2.4 mmol) of $Cd(OAc)_2 \cdot 2H_2O$, 1096 mg (4.8 mmol) of myristic acid, and 60 mL of ODE was heated to 130 °C in a three-neck flask upon bubbling with nitrogen flow for 1 h. Then, additional 60 mL of ODE were added, the flask was cooled down to 90 °C, and the mixture was degassed under vacuum for 30 min. After degassing, the reaction system was cooled down to 60 °C under stirring and N_2 flow, and 95 mg (1.2 mmol) of Se powder were added. The flask was quickly heated up to 240 °C and when the temperature reached 212 °C, a mixture of 166 mg (0.72 mmol) of $Cd(OAc)_2$ and 427 mg (1.6 mmol) of $Cd(OAc)_2 \cdot 2H_2O$ was added swiftly into the reaction solution. Upon reaching 240 °C, the solution was kept for 4 min at this temperature for the NPLs growth. Then, the heating mantle was removed, and when the temperature of the solution was decreased to 180 °C, 16 mL of OlAc were added, after which the reaction system was allowed to cool down to room temperature. The as-synthesized NPLs were precipitated by the addition of IPA with subsequent centrifugation. The supernatant was discarded, and the precipitate was dispersed in 22 mL of hexane and shaken for 40 min. The resulting solution was centrifuged for 5 min at 8000 rpm (6797 RCF) to remove byproduct 3 ML NPLs. The precipitate was discarded, and ethanol was added portion-wise until the solution became turbid. The mixture was centrifuged, the supernatant was discarded, and the precipitate was redispersed in 75 mL of ODE for the next synthesis step. Thus obtained NPLs in ODE, 533 mg (2 mmol) of $Cd(OAc)_2 \cdot 2H_2O$, and 0.5 mL of OlAc were loaded into a three-neck flask, degassed for 30 min at room temperature, and heated up to 200 °C under Ar flow. At this temperature, 0.2 mL of Se suspension (0.8 mmol of Se powder in 0.3 mL of OlAc and 2 mL of ODE) were added to the reaction system dropwise every 15 min (4–8 times depending on the desired lateral sizes of the NPLs; the suspension was properly shaken before each addition). After 15 min, the solution was cooled to room temperature and the NPLs were precipitated by adding IPA and centrifugation. The supernatant was discarded, and the precipitate was dispersed in chloroform for further use.

Cd^{2+}-to-Hg^{2+} CE in 4 ML CdSe NPLs[2]

For the synthesis of $Cd_xHg_{1-x}Se$ NPLs, CdSe NPLs (0.4 mmol of CdSe, concentration was determined optically[3]) were dispersed in 50 mL of chloroform followed by adding 9.4 mL of OlAm under stirring. Right after the addition of OlAm, 8 mL of the Hg precursor solution was injected. After 10 min, 0.7 mL of OlAc were added to the reaction mixture and it was left stirring at room temperature for 24 h in a properly closed flask. After 24 h, the mixture was centrifuged for 2 min at 4000 rpm (1699 RCF). The precipitate was discarded, and a mixture of acetonitrile and methanol (v/v = 4/1) was added portion-wise until the solution became turbid. The mixture was centrifuged again for 5 min at 10 000 rpm (10 621 RCF), the supernatant was discarded, and the precipitate was dispersed in 5 mL of pure chloroform and again centrifuged with acetonitrile. The final precipitate was dispersed in a mixture of 180 mL of ODE and 18 mL of OlAm for further shell growth (the growth was performed on freshly synthesized NPLs). The Hg precursor was prepared by dissolving 0.8 g (2.5 mmol) of $Hg(OAc)_2$ in a mixture of 3.7 mL of OlAm and 6.3 mL of chloroform at room temperature using ultrasonication. The precursor was used right after the preparation.

Synthesis of $Cd_xHg_{1-x}Se/Cd_yZn_{1-y}S$ core/shell NPLs[2]

The $Cd_xHg_{1-x}Se$ NPLs dispersion prepared as described above was loaded into a three-neck flask followed by the addition of 2.7 mL of OlAc, 0.32 g (1.40 mmol) of $Cd(OAc)_2$, and 3.47 g (9.6 mmol) of $Zn(DDTC)_2$. The reaction mixture was degassed for 40 min at room temperature and slowly heated (10 °C min^{-1}) up to 130 °C under Ar flow. After 10 min, the Ar flow was stopped, and the reaction mixture was stirred at 130 °C for 24 h. Then, the flask was cooled down to room temperature, the NPLs were precipitated by adding IPA with subsequent centrifugation. The precipitate was dissolved in 10 mL of chloroform and the solution was placed in a refrigerator overnight. Then, the solution was centrifuged for 2 min at 4000 rpm (1699 RCF). The white precipitate (unreacted salts) was discarded and the NPLs were precipitated from the supernatant using acetonitrile followed by centrifugation. The precipitate was dissolved in 10 mL of chloroform with 35 mg of $Zn(OAc)_2{\cdot}2H_2O$ and 25 mL of OlAc. The mixture was gently shaken overnight and then centrifuged for 3 min at 4000 rpm (1699 RCF) to separate undissolved $Zn(OAc)_2{\cdot}2H_2O$. Then, acetonitrile was added dropwise to the supernatant until the solution became turbid. The mixture was centrifuged for 3 min at 10 000 rpm (10 621 RCF), the supernatant was discarded, and the precipitate was dried under vacuum, weighed, and dissolved in chloroform with 10 mg mL^{-1} concentration.

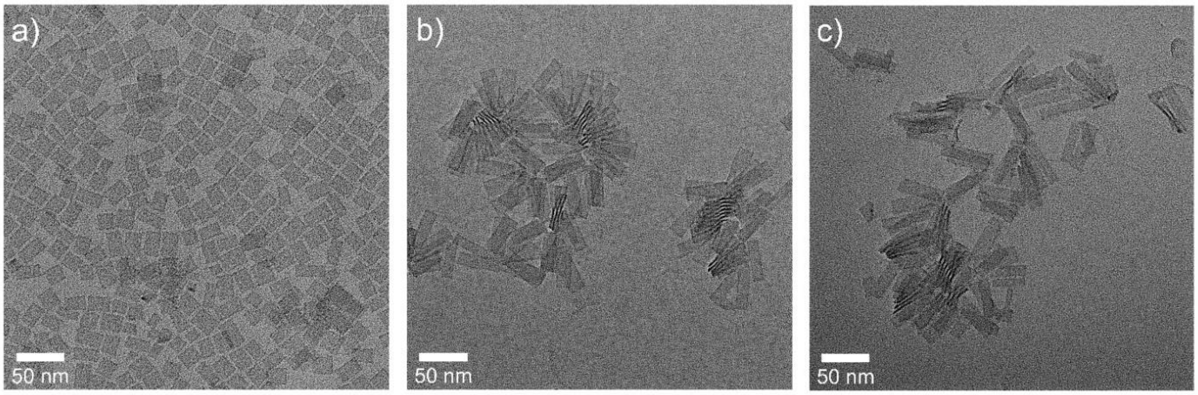

Figure S1. *TEM images of 4 ML-thick CdSe NPLs with lateral sizes of (a) 20.2 × 15.5 nm^2, (b) 41.2 x 14.5 nm^2, and (c) 47.0 x 13.8 nm^2, all used in the cation exchange reaction.*

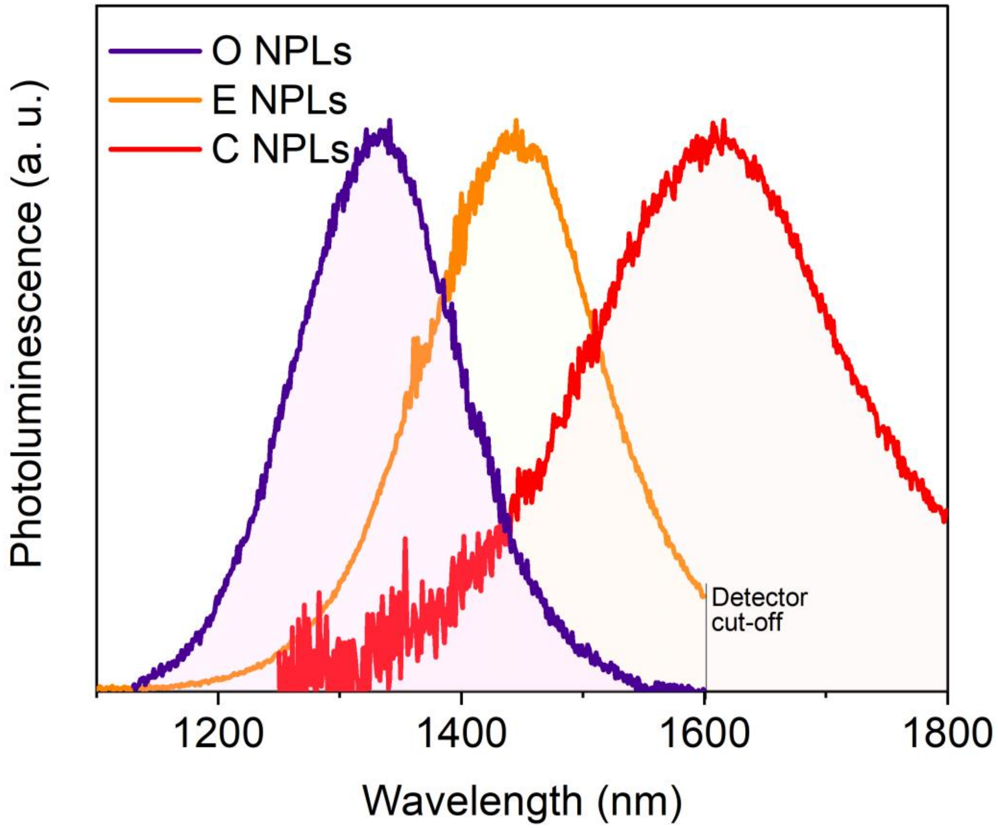

Figure S2. *PL spectra from solid films of O, E, and C NPL samples.*

Table S1. *Emission characteristics of all three samples, O, E, and C NPLs, in tetrachloroethylene (TCE) dispersions and in solid films obtained via spin-coating.*

	**TCE dispersions**			**Solid Films**		
**Sample**	**Peak PL**	**FWHM**	**PLQY (%)**	**Peak PL**	**FWHM**	**PLQY (%)**
**O NPLs**	1297 nm (0.956 eV)	159 nm (0.118 eV)	58±6	1335 nm (0.929 eV)	160 nm (0.112 eV)	39±4
**E NPLs**	1416 nm (0.876 eV)	180 nm (0.112 eV)	54±5	1444 nm (0.859 eV)	180 nm (0.107 eV)	35±3
**C NPLs**	1566 nm (0.792 eV)	241 nm (0.123 eV)	50±5	1608 nm (0.771 eV)	262 nm (0.126 eV)	30±3

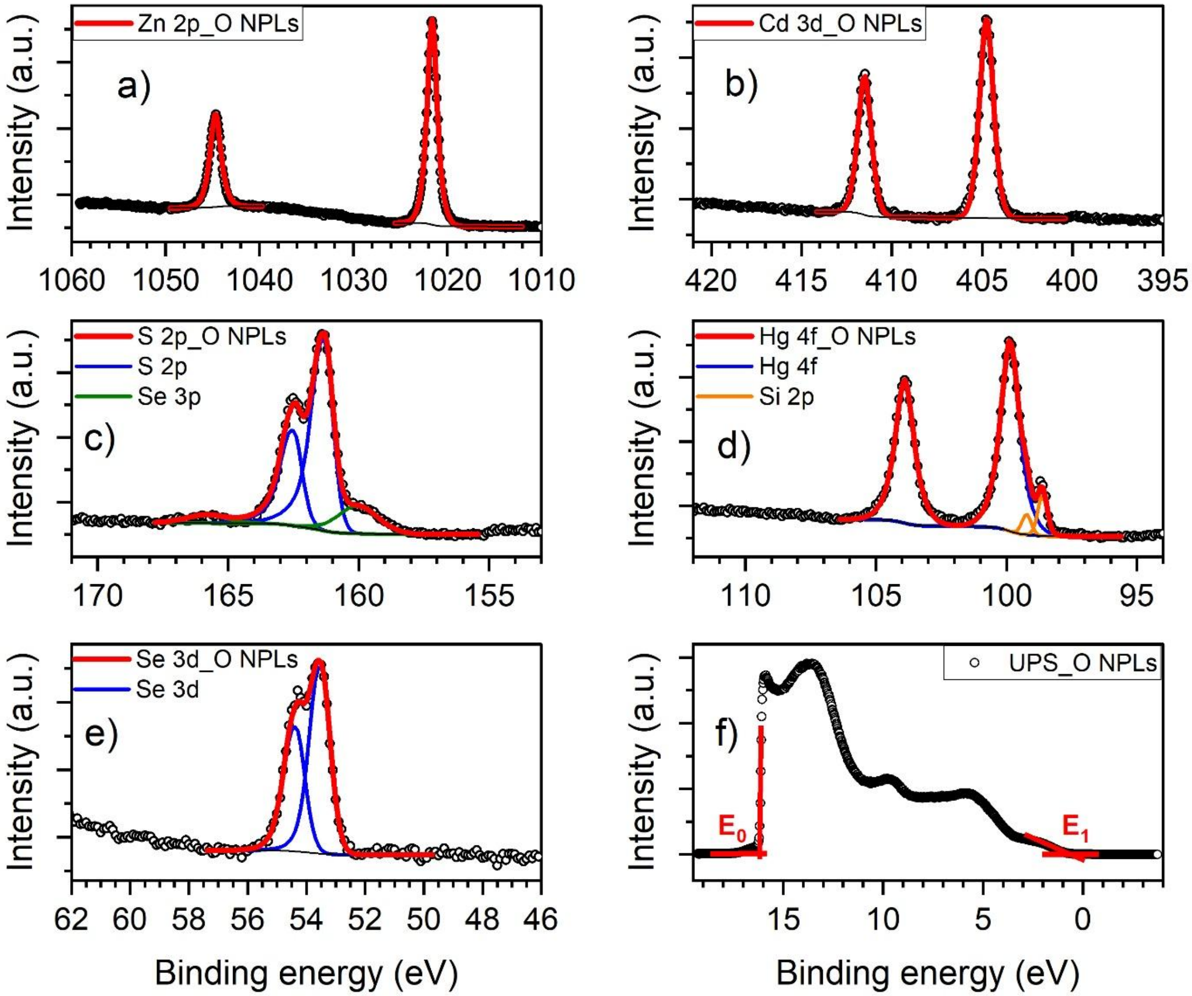

Figure S3. *XPS and UPS analyses of O NPL sample. High-resolution XPS core-level signals of a) Zn 2p, b) Cd 3d, c) S 2p (the Se 3p doublet, which stays in this energy range, was deconvoluted from the S 2p peak), d) Hg 4f (the Si 2p doublet, due to silicon substrate, stays in this energy range, and was deconvoluted from the Hg 4f peak), and e) Se 3d. f) UPS spectrum of the sample, in which the positions of E_0 and E_1 are reported.*

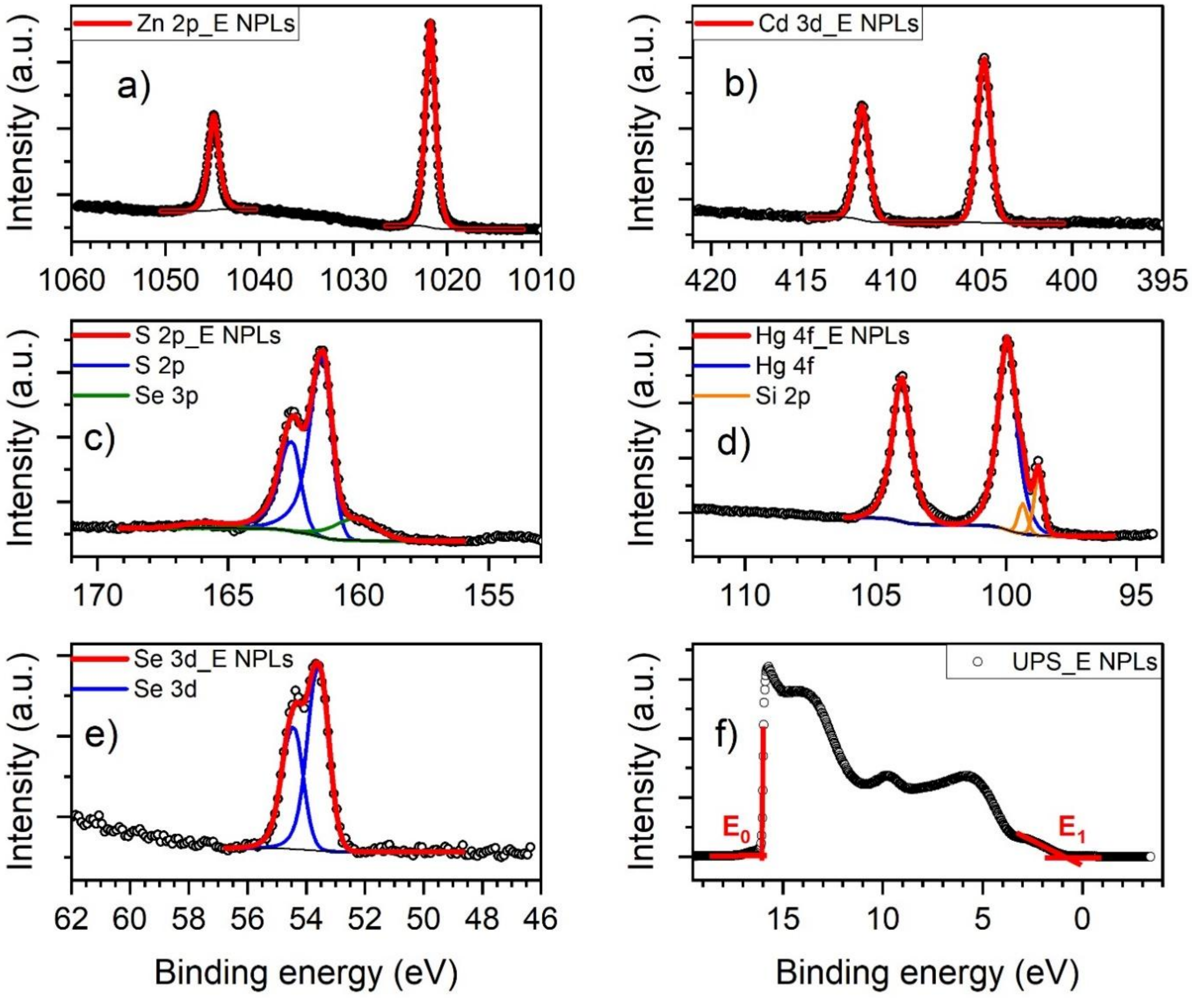

Figure S4. *XPS and UPS analyses of E NPL sample. High-resolution XPS core-level signals of a) Zn 2p, b) Cd 3d, c) S 2p (the Se 3p doublet, which stays in this energy range, was deconvoluted from the S 2p peak), d) Hg 4f (the Si 2p doublet, due to silicon substrate, stays in this energy range, and was deconvoluted from the Hg 4f peak), and e) Se 3d. f) UPS spectrum of the sample, in which the positions of E_0 and E_1 are reported.*

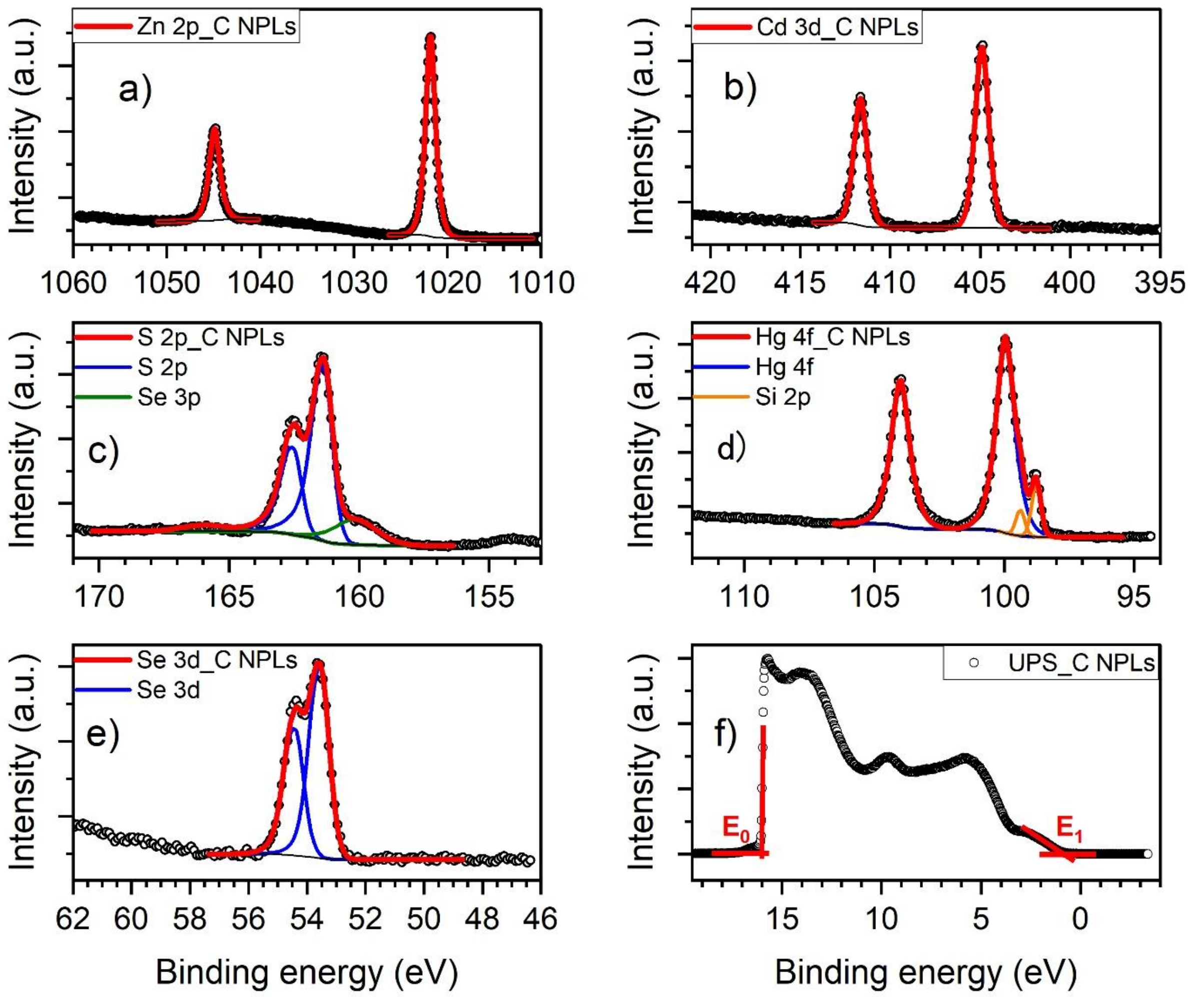

Figure S5. *XPS and UPS analyses of C NPL sample. High-resolution XPS core-level signals of a) Zn 2p, b) Cd 3d, c) S 2p (the Se 3p doublet, which stays in this energy range, was deconvoluted from the S 2p peak), d) Hg 4f (the Si 2p doublet, due to silicon substrate, stays in this energy range, and was deconvoluted from the Hg 4f peak), and e) Se 3d. f) UPS spectrum of the sample, in which the positions of E_0 and E_1 are reported.*

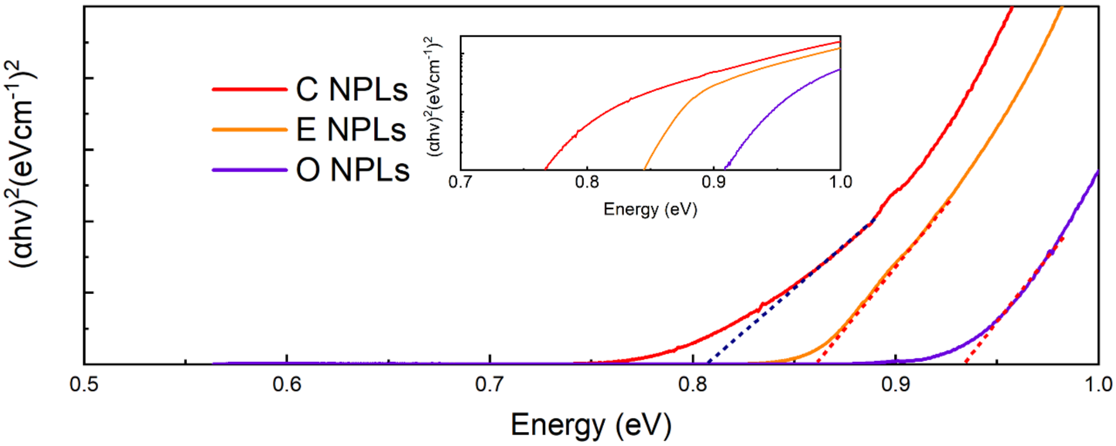

Figure S6. *Tauc plots of samples O, E, and C over linear and logarithmic (inset image) scale.*

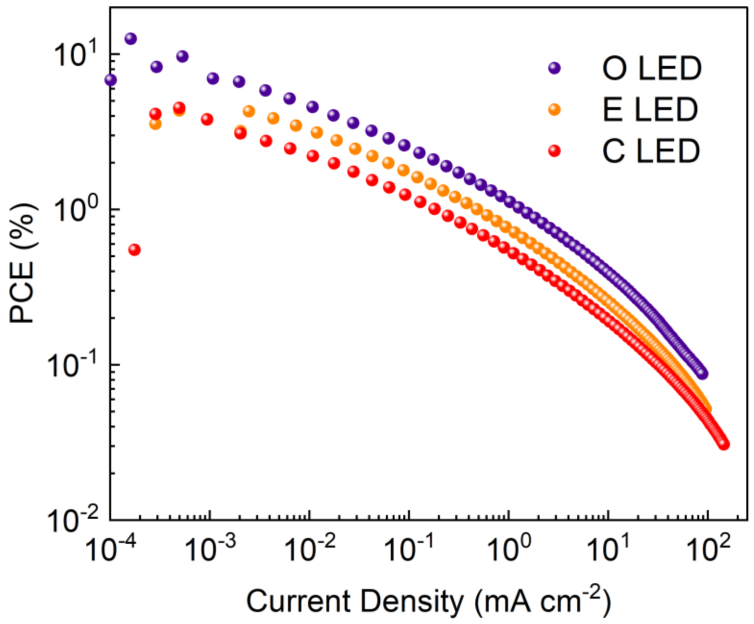

Figure S7. *Power conversion efficiency (PCE) versus current density curves for the LEDs.*

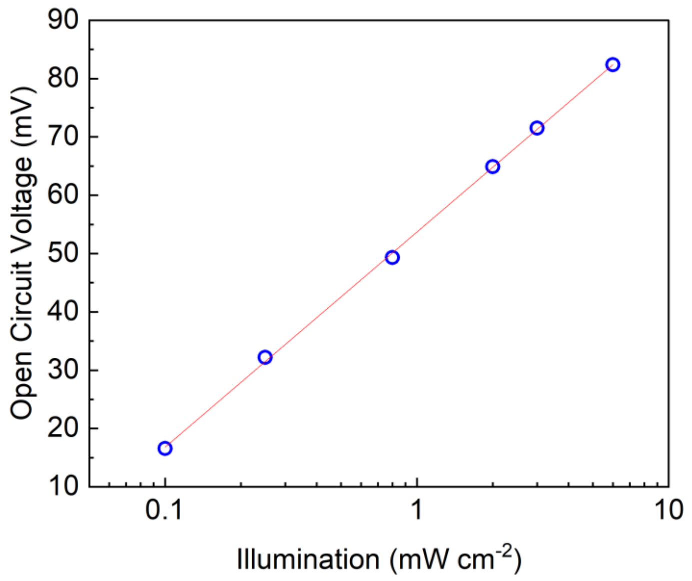

Figure S8. *Open circuit voltage of the photodiode versus illumination intensity (1200 nm).*

Table S2. *Comparison between the photodiode of this work and state-of-art devices based on other colloidal semiconductor nanocrystals.*

**Structure**	**J_d (A)**	**Bias (V)**	**EQE (%)**	**Responsivity (A/W)**	**Wavelength (nm)**	**ref**
$TiO_2/InSb/MoO_x$<br>$ZnO/InSb/MoO_x$	-	0	5<br>0.6	-	1240	[4]
$ZnO/InSb/MoO_x$	-	0	2.7	-	1200	[5]
$NiO_x/PbS/PbS$-EDT	10^{-7}	0	25	0.25	1370	[6]
NiO_x/PbS CQD/C_{60}/ZnO	17.8×10^{-9}	0.5<br>0	60<br>28	0.4<br>0.2	970<br>970	[7]
AZO/PbS CQDs/Si		7	33	0.4	1230	[8]
p-Si/SiN_x/PbS–TBAI	6×10^{-6}	0.05	27.5	-	1310	[9]
ZnO/PbS–PbX_2/PbS–BA	5.2×10^{-8}	-	80	-	1230	[10]
ZnO/CdHgSe/MoO_x	2.22×10^{-8}	0	25	0.241	1200	This Work

References

(1) Mitrofanov, A.; Prudnikau, A.; Di Stasio, F.; Weiß, N.; Hübner, R.; Dominic, A. M.; Borchert, K. B. L.; Lesnyak, V.; Eychmüller, A. Near-Infrared-Emitting $Cd_xHg_{1-x}Se$-Based Core/Shell Nanoplatelets. *Chem. Mater.* **2021**, *33* (19), 7693–7702. https://doi.org/10.1021/acs.chemmater.1c01682.

(2) Prudnikau, A.; Roshan, H.; Paulus, F.; Martín-García, B.; Hübner, R.; Bahmani Jalali, H.; De Franco, M.; Prato, M.; Di Stasio, F.; Lesnyak, V. Efficient Near-Infrared Light-Emitting Diodes Based on CdHgSe Nanoplatelets. *Adv. Funct. Mater.* **2024**, *34,* 2310067. https://doi.org/10.1002/adfm.202310067.

(3) Achtstein, A. W.; Antanovich, A.; Prudnikau, A.; Scott, R.; Woggon, U.; Artemyev, M. Linear Absorption in CdSe Nanoplates: Thickness and Lateral Size Dependency of the Intrinsic Absorption. *J. Phys. Chem. C* **2015**, *119* (34), 20156–20161. https://doi.org/10.1021/acs.jpcc.5b06208.

(4) Peng, L.; Wang, Y.; Ren, Y.; Wang, Z.; Cao, P.; Konstantatos, G. InSb/InP Core–Shell Colloidal Quantum Dots for Sensitive and Fast Short-Wave Infrared Photodetectors. *ACS Nano* **2024**, *18* (6), 5113–5121. https://doi.org/10.1021/acsnano.3c12007.

(5) Muhammad; Choi, D.; Parmar, D. H.; Rehl, B.; Zhang, Y.; Atan, O.; Kim, G.; Xia, P.; Pina, J. M.; Li, M.; Liu, Y.; Voznyy, O.; Hoogland, S.; Sargent, E. H. Halide-Driven Synthetic Control of InSb Colloidal Quantum Dots Enables Short-Wave Infrared Photodetectors. *Adv. Mater.* **2023**, *35*, 2306147. https://doi.org/10.1002/adma.202306147.

(6) Wang, X.; Song, Z.; Tang, H.; Li, Y.; Zhong, H.; Wu, J.; Wang, W.; Chen, S.; Zhang, W.; Fang, F.; Hao, J.; Wu, D.; Müller-Buschbaum, P.; Cao, L.; Tang, Z.; Tang, J.; Zhang, L.; Wang, K.; Chen, W. Synergic Surface Modifications of PbS Quantum Dots by Sodium Acetate in Solid-State Ligand Exchange toward Short-Wave Infrared Photodetectors. *ACS Appl. Mater. Interfaces* **2024,** *16* (33), 44164–44173. https://doi.org/10.1021/acsami.4c05201.

(7) Liu, J.; Liu, P.; Chen, D.; Shi, T.; Qu, X.; Chen, L.; Wu, T.; Ke, J.; Xiong, K.; Li, M.; Song, H.; Wei, W.; Cao, J.; Zhang, J.; Gao, L.; Tang, J. A Near-Infrared Colloidal Quantum Dot Imager with Monolithically Integrated Readout Circuitry. *Nat. Electron.* **2022**, *5* (7), 443–451. https://doi.org/10.1038/s41928-022-00779-x.

(8) Masala, S.; Adinolfi, V.; Sun, J.; Gobbo, S. Del; Voznyy, O.; Kramer, I. J.; Hill, I. G.; Sargent, E. H. The Silicon:Colloidal Quantum Dot Heterojunction. *Adv. Mater.* **2015**, *27* (45), 7445–7450. https://doi.org/10.1002/adma.201503212.

(9) Wang, J.; Chen, J. High-Sensitivity Silicon: PbS Quantum Dot Heterojunction near-Infrared Photodetector. *Surf. Interfaces* **2022**, *30*, 101945. https://doi.org/10.1016/j.surfin.2022.101945.

(10) Lee, S.; Choi, M.-J.; Sharma, G.; Biondi, M.; Chen, B.; Baek, S.-W.; Najarian, A. M.; Vafaie, M.; Wicks, J.; Sagar, L. K.; Hoogland, S.; de Arquer, F. P. G.; Voznyy, O.; Sargent, E. H. Orthogonal Colloidal Quantum Dot Inks Enable Efficient Multilayer Optoelectronic Devices. *Nat. Commun.* **2020**, *11* (1), 4814. https://doi.org/10.1038/s41467-020-18655-7.
